# New Sources, Differentiation, and Therapeutic Uses of Mesenchymal Stem Cells

**DOI:** 10.3390/ijms22105288

**Published:** 2021-05-18

**Authors:** Saeyoung Park, Sung-Chul Jung

**Affiliations:** 1 Departments of Biochemistry, College of Medicine, Ewha Womans University, Seoul 07804, Korea; saeyoung@ewha.ac.kr; 2 Graduate Program in System Health Science and Engineering, Ewha Womans University, Seoul 03760, Korea

Mesenchymal stem cells (MSCs) are multipotent cells derived from various tissues including bone marrow and adipose tissues. MSCs have the capacity to differentiate into mesodermal lineages, including chondroblasts, osteoblasts, and adipocytes. In addition to bone marrow and adipose tissues, Wharton’s jelly, umbilical cord, fetal/neonatal tissues [1], dental pulp [2], and placenta [3] have been studied as sources of MSCs that can differentiate into various cell types with therapeutic properties. The clinical applications of MSCs are based on their unique stem cell properties, including the secretion of trophic factors and their proangiogenic, anti-inflammatory, immune-modulatory, and anti-oxidative stress activities. However, large-scale expansion of these cells for allogeneic therapies requires minimization of donor-dependent and bioprocess variabilities [4]. This Special Issue, entitled “New Sources, Differentiation, and Therapeutic Uses of Mesenchymal Stem Cells”, includes eight articles, four of which are review papers that discuss novel sources of MSCs and recent advances in the characterization and applications of MSCs. The articles in this issue provide insight into the therapeutic uses of MSCs and their derivatives, such as extracellular vesicles (EVs) and MSC spheroids.

Menstrual blood [5], tonsils [6], and induced pluripotent stem cells (iPSCs) [7] are also attracting attention as novel tissue sources for MSCs and are expected to be suitable as cell therapy products. The iPSC-derived MSCs have been applied to skin regeneration and skin rejuvenation [8,9], and menstrual blood-derived MSCs (MB-MSCs) have shown angiogenic potential similar to that of bone marrow-derived MSCs (BM-MSCs) [5,10]. Tonsil-derived stem cells have excellent proliferation and differentiation capabilities, and their clinical applications as therapeutic agents have been studied [6,11,12].

Over the past decade, MSCs have been proposed as a promising therapeutic treatment for various diseases. Many preclinical and clinical studies have described various strategies for effective MSC therapy, including decisions about the most (1) satisfactory cell type for each therapeutic application, (2) satisfactory culture conditions to ensure therapeutic effects, (3) suitable and effective methods for the mass production of these cells, and (4) appropriate functional tests for determining whether these biological products for each therapeutic indication have been developed to overcome the limitations of MSCs, such as heterogeneity and safety and handling issues [4].

To optimize the clinical applications, the approaches used to develop biological products based on the molecular properties of MSCs and their mechanisms of action are being studied. Among these approaches, the paracrine function of MSCs via the secretome, which involves conditioned media (CM), EVs, and exosomes, is considered to be representative [5,9,13,14,15,16,17]. The CM derived from BM-MSCs and MB-MSCs have been shown to be capable of stimulating tube-like formation of human umbilical vein endothelial cells [5]. CM derived from BM-MSCs, amniotic membrane MSCs, umbilical cord blood MSCs, and umbilical cord tissue MSCs (UC-MSCs) have been shown to be effective treatments in rodent models of bronchopulmonary dysplasia (BPD) [15]. Ramalingam et al. reported the therapeutic role of CM derived from neural-induced adipose tissue-derived MSCs (AD-MSCs) against rotenone-induced Parkinson’s disease-like impairments [14].

EVs of the MSC secretome can generate an encouraging alternative for exploiting MSC properties and can be classified as exosomes (30–120 nm in diameter), which originate within endosomal compartments called multivesicular bodies in the cell [4]. EVs from AD-MSCs have anti-photoaging potential and have been used in subcutaneous injections in mouse models of photoaging [9,17]. In addition, the capacity to inhibit inflammation, which is consistent with the main actions described for EVs in general [15,18], has been observed in animal BPD models. Transmission of cellular senescence and proinflammatory activation between MSCs and their EVs are involved in the development of inflamm-aging, which is associated with the degeneration of organs and tissues during aging [16]. Mato-Basalo et al. reported that treatment of senescent UC-MSCs with small inhibitors (e.g., JSH-23, MG-132, or curcumin) prevented cellular senescence and proinflammatory activation in MSCs, and paracrine and proinflammatory transmission by EVs through inhibition of the p65 pathway [16].

To advance the development of innovative stem cell therapies, priming [15,16,17,18,19] or genetic engineering of MSCs and biomaterial-based physical/structural modification [5,13,15] of MSCs have been studied. Treatment of AD-MSCs with fibronectin-derived peptide has been shown to improve their proliferation and differentiation into osteoblasts [19]. An improved therapeutic effect of BM-MSCs treated with recombinant erythropoietin in a rodent BPD model has also been reported [15,20]. In addition, genetic engineering techniques have been applied to induce insufficient endogenous factors or new proteins directly within MSCs [21]. Various MSCs have been used with genetic modification technology using RNA viruses, such as lentiviruses and retroviruses, and DNA viruses, including adenoviruses or adeno-associated viruses, and the preclinical results of these studies have been published [4]. The formation of spheroids that recover cell communication and provide a concertation gradient of external factors depending on the location, as observed in vivo, has been reported. These spheroids exhibit superior viability, self-renewal capacity, and differentiation potential compared with two-dimensional cells [13].

As presented in this issue, several biotechnology techniques have been developed to overcome the limitations noted in previous reports on the clinical applications of MSCs and to produce high-efficiency MSCs. MSCs and their products applied using these biotechnology techniques should focus on standardization to ensure the safety verification and cell quality control needed for practical clinical applications. The accumulated results of these studies will ultimately accelerate the development and practical clinical applications of high-efficiency MSCs and their product therapeutics.
